# The Atlanta Medical Society

**Published:** 1882-04

**Authors:** 


### Societies.

### ATLANTA MEDICAL SOCIETY.

*Reported for the Atlanta Medical Register.*

FEBRUARY 18th, 1882.

The President, Dr. W. S. Armstrong, in the chair.

Dr. James B. Baird, read a paper on vaccination. (See page 385).

There was some discussion of the subject of the essay, the members, in the main, endorsing the views as put forth by the essayist.

Dr. J. R. Stodghill, recently engaged in the vaccination department conducted by the Board of Health, stated that of three thousand vaccinations, he had seen severe inflammation or other trouble in only three or four. These were cases of arm to arm vaccination. Had vaccinated one hundred persons from one subject, who had three vesicles. Always vaccinates in six different points.

Dr. W. D. Bizzell stated that in the epidemic in Mobile in 1875, there were seven men confined in one cell of the jail, and forty or fifty in other portions of the prison. One man in the cell developed small-pox; after three days of fever, preceding the eruption, he was removed, and finally died of the malignant form of the disease. The others had a change of clothing, the old ones and bedding were burned, the skin thoroughly washed with carbolic acid, and the cell closed, fumigated with burning sulphur, and then whitewashed. All the prisoners were vaccinated, four places in each arm of each patient. Only one prisoner contracted the disease. This one was in a cell several doors from the one where the disease first appeared. All the inmates of this first cell escaped the contagion. This patient was also removed, and died. The vaccination did not *take* on this man. Dr. B. afterward learned that immediately after the vaccination, he had washed the virus from the wound.

Dr. Taliaferro believes that the bovine will take as often as the humanized crust, if rightly applied. Having observed the violence of certain cutaneous diseases, as occurring in patients recently vaccinated, is rather of the opinion that it is inadvisable to vaccinate during epidemics of measles, etc.

Dr. F. H. O'Brien had observed one case of eczema in a child evidently aggravated and prolonged by vaccination. Preferred bovine to humanized virus, as more convenient and just as reliable. He once vaccinated four children who had had small-pox, and were well pitted. In one, only six months had elapsed after the disease; first vaccination failed; the second, a week later, was entirely successful. No success attended the vaccination of the second child; two years had passed since the attack of small-pox. The vaccination was successful in the third and fourth children. Several years had passed since they had the small-pox.

Dr. J. G. Earnest thinks that severe skin diseases contraindicate vaccination, unless in emergency. Prefers bovine to humanized virus.

Dr. J. T. Johnson thought the bovine not superior to the humanized virus, where the fresh lymph or the fresh crust (of dessicated lymph) is used. Bovine often fails because of insufficient application. Thinks it well to use it in revaccinations. Has seen rather severe results follow its use; in one case the whole arm was inflamed, and dotted with numerous characteristic vaccine vesicles. Believes, contrary to the now fashionable idea, that multiple punctures are not more efficient than single ones, further than better insuring absorption. Multiple "scars" may be better evidence than single ones, if the result of successive vaccinations.

Dr. Stodghill had successfully vaccinated several persons who claimed to have had variola.

---

March 4th, 1882.

Dr. W. S. Armstrong presiding.

Dr. G. G. Roy related an interesting case of monstrosity, or deformed fœtus, which he witnessed some years since. The abortion occurred in a few weeks after conception and was produced by accident. Ten weeks later another fœtus, five or six months advanced, well-formed, was delivered by the same patient. Dr. R. also reported a case occurring recently in his practice probably showing arrested development of the fœtus. It was apparently but a few weeks advanced. Three hours after this another one of six months was born. The doctor believed, however, that it is possible for conception to occur in a womb already impregnated; and instanced some of the lower animals, as the dog, in which it often was evident that the litter is the offspring of several different fathers.

Dr. J. B. Baird said that this might occur also in the human female, if connection was had with two different males with but a short interval of time. Otherwise, such impregnation is probably impossible. Instances seeming to show the contrary, are cases of arrest of development of one fœtus.

Dr. Roy referred to a case of a woman who cherished a strong dislike for a club-footed man, and frequently imitated his gait. Before this she had borne three children, properly formed. Afterward, she bore three, all deformed by clubbed feet.

Dr. Connally mentioned the case of a woman in Alabama, who while pregnant stepped out into the yard about twilight, and became frightened at the vision of a dressed (opened) hog hanging there. The child all through life had a raw-looking strip down the front of the integument of the chest and abdomen. He is now an old man, and the strip is a discharging surface.

Dr. Baird desired again to call attention to the importance of comparing clinical thermometers with some accurate standard. Knowing that his was not entirely accurate, he had recently compared it with the Yale standard, and found it one-and-a-half degrees too high—varying about the same extent for the different temperatures.

Dr. Baird also related the details of a case in which a threaded needle became embedded, eyed-end foremost, in the arm of a little girl five years of age. Moderate traction on the thread failed to move it. After some hesitation he decided to allow it to remain for a time, even though the thread might have been used as a guide for the incision in removing it. Nine days later, suppuration had occurred, and the needle was easily removed by drawing on the thread.

Drs. Armstrong and Johnson each thought the delay was justifiable; and spoke, also, of the advisability, ordinarily, of not cutting for needless, or pieces, unless they could be certainly located. Dr. A. said that a few days since he had declined to cut for a needle in the person of a lady. Another physician, advising differently, consumed three hours in an unsuccessful search for it.

Dr. Roy spoke of a man who swallowed a needle and thread (he was holding the threaded end in his mouth); two years later, after a violent pain at stool, the needle was discharged *per anum*.

